# 4-(Dimethyl­amino)phenyl phenyl ketone

**DOI:** 10.1107/S1600536808020953

**Published:** 2008-07-12

**Authors:** Hoong-Kun Fun, Samuel Robinson Jebas

**Affiliations:** a X-ray Crystallography Unit, School of Physics, Universiti Sains Malaysia, 11800 USM, Penang, Malaysia

## Abstract

In the crystal structure of the title compound, C_15_H_15_NO, the two benzene rings are twisted from each other by a dihedral angle of 47.97 (4)°. The crystal structure is stabilized by weak inter­molecular C—H⋯O and C—H⋯π inter­actions, and π–π inter­actions with a centroid–centroid distance of 3.8493 (5) Å are observed.

## Related literature

For related literature on non-linear optical properties of benzophenone, see: Arivanandhan *et al.* (2006 ▶); Szyrszyng *et al.* (2004 ▶); Vijayan *et al.* (2002 ▶) & Wang *et al.*, (2007 ▶). For bond-length data see: Allen *et al.* (1987 ▶)
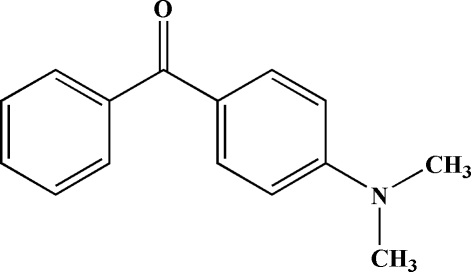


## Experimental

### 

#### Crystal data

C_15_H_15_NO
*M* *_r_* = 225.28
Monoclinic, 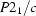

*a* = 13.0575 (3) Å
*b* = 7.7456 (2) Å
*c* = 12.4931 (3) Å
β = 111.717 (1)°
*V* = 1173.85 (5) Å^3^
*Z* = 4
Mo *K*α radiation
μ = 0.08 mm^−1^
*T* = 100.0 (1) K
0.60 × 0.43 × 0.28 mm

#### Data collection

Bruker SMART APEXII CCD area-detector diffractometer
Absorption correction: multi-scan (*SADABS*; Bruker, 2005 ▶) *T*_min_ = 0.939, *T*_max_ = 0.977
22302 measured reflections
5156 independent reflections
4138 reflections with *I* > 2σ(*I*)
*R*_int_ = 0.028

#### Refinement

*R*[*F* ^2^ > 2σ(*F* ^2^)] = 0.047
*wR*(*F* ^2^) = 0.138
*S* = 1.06
5156 reflections
156 parameters
H-atom parameters constrained
Δρ_max_ = 0.42 e Å^−3^
Δρ_min_ = −0.25 e Å^−3^

### 

Data collection: *APEX2* (Bruker, 2005 ▶); cell refinement: *APEX2*; data reduction: *SAINT* (Bruker, 2005 ▶); program(s) used to solve structure: *SHELXTL* (Sheldrick, 2008 ▶); program(s) used to refine structure: *SHELXTL*; molecular graphics: *SHELXTL*; software used to prepare material for publication: *SHELXTL* and *PLATON* (Spek, 2003 ▶).

## Supplementary Material

Crystal structure: contains datablocks global, I. DOI: 10.1107/S1600536808020953/at2585sup1.cif

Structure factors: contains datablocks I. DOI: 10.1107/S1600536808020953/at2585Isup2.hkl

Additional supplementary materials:  crystallographic information; 3D view; checkCIF report

## Figures and Tables

**Table 1:** Hydrogen-bond geometry (Å, °)

*D*—H⋯*A*	*D*—H	H⋯*A*	*D*⋯*A*	*D*—H⋯*A*
C4—H4⋯O1^i^	0.93	2.46	3.3730 (12)	168
C10—H10⋯*Cg*2^ii^	0.93	2.98	3.6452 (9)	130

Symmetry codes: (i) 
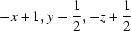
; (ii) 
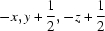
. *Cg*2 is the centroid of atoms C8–C13.

**Fig. 1.**
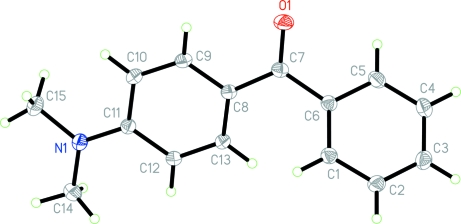
The molecular structure of the title compound, showing 50% probability displacement ellipsoids and the atom numbering scheme.

**Fig. 2.**
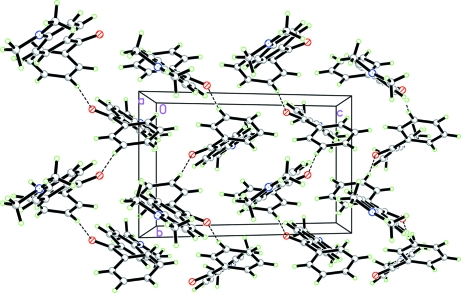
The crystal packing of the title compound, viewed along the a axis. Hydrogen bonds are shown as dashed lines.

## supplementary crystallographic information

### Comment

Benzophenone and its derivatives exhibits non-linear optical properties (Wang *et al.*, 2007; Vijayan *et al.*, 2002 & Arivanandhan *et al.*, 2006) and are good candidates for the non-linear optical applications (Szyrszyng *et al.*, 2004). In view of the importance of the benzophenone derivatives, the crystal structure of the title compound (I) has been elucidated.

The asymmetric unit of (I) consists of one molecule of 4-(dimethylamino)benzophenone. Bond lengths and angles in the molecule are found to have normal values (Allen *et al.*, 1987) The dihedral angle formed by the rings (C1–C6) and (C8–C13) is 47.97 (4)° indicating that the rings are twisted from each other. The crystal packing (Fig.2) is consolidated by intermolecular C—H···O hydrogen bonds and C—H···π interactions. π–π interactions with the centroid to centroid distance of 3.8493 (5)Å are observed.

### Experimental

4-(Dimethylamino)benzophenone was purchased from Aldrich and dissolved in ethanol. The solution was allowed to evaporate slowly. Colourless crystals were obtained after a month.

### Refinement

H atoms were positioned geometrically [C—H = 0.93Å and CH_3_=0.96 Å] and refined using a riding model, with *U*_iso_(H) = 1.2 or 1.5*U*_eq_(C). The rotating group model was considered for the methyl H atoms.

### Crystal data

**Table:**

C_15_H_15_NO	*F*_000_ = 480
*M**_r_* = 225.28	*D*_x_ = 1.275 Mg m^−^^3^
Monoclinic, *P*2_1_/*c*	Mo *K*α radiation λ = 0.71073 Å
Hall symbol: -P 2ybc	Cell parameters from 8685 reflections
*a* = 13.0575 (3) Å	θ = 3.1–38.7º
*b* = 7.7456 (2) Å	µ = 0.08 mm^−^^1^
*c* = 12.4931 (3) Å	*T* = 100.0 (1) K
β = 111.7170 (10)º	Block, colourless
*V* = 1173.85 (5) Å^3^	0.60 × 0.43 × 0.28 mm
*Z* = 4	

### Data collection

**Table:**

Bruker SMART APEXII CCD area-detector diffractometer	5156 independent reflections
Radiation source: fine-focus sealed tube	4138 reflections with *I* > 2σ(*I*)
Monochromator: graphite	*R*_int_ = 0.028
*T* = 100.0(1) K	θ_max_ = 35.0º
φ and ω scans	θ_min_ = 3.1º
Absorption correction: multi-scan(SADABS; Bruker, 2005)	*h* = −21→19
*T*_min_ = 0.939, *T*_max_ = 0.977	*k* = −10→12
22302 measured reflections	*l* = −20→20

### Refinement

**Table:**

Refinement on *F*^2^	Secondary atom site location: difference Fourier map
Least-squares matrix: full	Hydrogen site location: inferred from neighbouring sites
*R*[*F*^2^ > 2σ(*F*^2^)] = 0.047	H-atom parameters constrained
*wR*(*F*^2^) = 0.138	*w* = 1/[σ^2^(*F*_o_^2^) + (0.0744*P*)^2^ + 0.1876*P*] where *P* = (*F*_o_^2^ + 2*F*_c_^2^)/3
*S* = 1.06	(Δ/σ)_max_ < 0.001
5156 reflections	Δρ_max_ = 0.42 e Å^−^^3^
156 parameters	Δρ_min_ = −0.25 e Å^−^^3^
Primary atom site location: structure-invariant direct methods	Extinction correction: none

### Special details

**Table:**

Experimental. The data was collected with the Oxford Cyrosystem Cobra low-temperature attachment.
Geometry. All e.s.d.'s (except the e.s.d. in the dihedral angle between two l.s. planes) are estimated using the full covariance matrix. The cell e.s.d.'s are taken into account individually in the estimation of e.s.d.'s in distances, angles and torsion angles; correlations between e.s.d.'s in cell parameters are only used when they are defined by crystal symmetry. An approximate (isotropic) treatment of cell e.s.d.'s is used for estimating e.s.d.'s involving l.s. planes.
Refinement. Refinement of *F*^2^ against ALL reflections. The weighted *R*-factor *wR* and goodness of fit *S* are based on *F*^2^, conventional *R*-factors *R* are based on *F*, with *F* set to zero for negative *F*^2^. The threshold expression of *F*^2^ > σ(*F*^2^) is used only for calculating *R*-factors(gt) *etc*. and is not relevant to the choice of reflections for refinement. *R*-factors based on *F*^2^ are statistically about twice as large as those based on *F*, and *R*- factors based on ALL data will be even larger.

### Fractional atomic coordinates and isotropic or equivalent isotropic displacement parameters (Å^2^)

**Table:**

	*x*	*y*	*z*	*U*_iso_*/*U*_eq_	
O1	0.26958 (5)	0.42158 (10)	0.20400 (5)	0.02533 (15)	
N1	−0.10640 (5)	0.34279 (10)	0.40740 (6)	0.01934 (14)	
C1	0.43352 (6)	0.31796 (11)	0.49440 (7)	0.01861 (15)	
H1	0.3933	0.3765	0.5308	0.022*	
C2	0.53797 (7)	0.25436 (12)	0.55889 (7)	0.02153 (16)	
H2	0.5677	0.2714	0.6382	0.026*	
C3	0.59761 (7)	0.16567 (12)	0.50493 (8)	0.02318 (17)	
H3	0.6668	0.1215	0.5484	0.028*	
C4	0.55457 (7)	0.14235 (12)	0.38612 (8)	0.02373 (17)	
H4	0.5948	0.0833	0.3500	0.028*	
C5	0.45115 (7)	0.20786 (11)	0.32186 (7)	0.02078 (16)	
H5	0.4228	0.1942	0.2423	0.025*	
C6	0.38907 (6)	0.29412 (10)	0.37532 (7)	0.01685 (14)	
C7	0.27935 (6)	0.36465 (10)	0.29960 (7)	0.01733 (14)	
C8	0.18415 (6)	0.36068 (10)	0.33575 (6)	0.01575 (14)	
C9	0.08856 (6)	0.44970 (10)	0.26790 (7)	0.01806 (15)	
H9	0.0897	0.5141	0.2055	0.022*	
C10	−0.00688 (6)	0.44473 (11)	0.29074 (7)	0.01847 (15)	
H10	−0.0687	0.5052	0.2436	0.022*	
C11	−0.01199 (6)	0.34866 (10)	0.38509 (6)	0.01540 (14)	
C12	0.08462 (6)	0.25969 (10)	0.45427 (6)	0.01625 (14)	
H12	0.0843	0.1963	0.5174	0.019*	
C13	0.17938 (6)	0.26547 (10)	0.42967 (6)	0.01610 (14)	
H13	0.2414	0.2051	0.4763	0.019*	
C14	−0.11461 (7)	0.24089 (12)	0.50120 (7)	0.02242 (16)	
H14A	−0.0465	0.2479	0.5664	0.034*	
H14B	−0.1292	0.1227	0.4773	0.034*	
H14C	−0.1735	0.2846	0.5220	0.034*	
C15	−0.20773 (7)	0.41652 (14)	0.32699 (8)	0.02666 (19)	
H15A	−0.1986	0.5387	0.3213	0.040*	
H15B	−0.2666	0.3950	0.3538	0.040*	
H15C	−0.2250	0.3646	0.2526	0.040*	

### Atomic displacement parameters (Å^2^)

**Table:**

	*U*^11^	*U*^22^	*U*^33^	*U*^12^	*U*^13^	*U*^23^
O1	0.0234 (3)	0.0354 (4)	0.0184 (3)	−0.0007 (2)	0.0091 (2)	0.0074 (2)
N1	0.0154 (3)	0.0231 (3)	0.0193 (3)	0.0014 (2)	0.0062 (2)	0.0022 (2)
C1	0.0176 (3)	0.0211 (3)	0.0172 (3)	−0.0011 (3)	0.0065 (3)	−0.0014 (3)
C2	0.0176 (3)	0.0274 (4)	0.0181 (3)	−0.0012 (3)	0.0049 (3)	0.0009 (3)
C3	0.0168 (3)	0.0271 (4)	0.0261 (4)	0.0013 (3)	0.0086 (3)	0.0050 (3)
C4	0.0208 (3)	0.0280 (4)	0.0262 (4)	0.0017 (3)	0.0133 (3)	0.0007 (3)
C5	0.0204 (3)	0.0254 (4)	0.0190 (3)	−0.0013 (3)	0.0102 (3)	−0.0012 (3)
C6	0.0162 (3)	0.0185 (3)	0.0166 (3)	−0.0019 (2)	0.0070 (2)	0.0003 (2)
C7	0.0183 (3)	0.0180 (3)	0.0156 (3)	−0.0021 (2)	0.0062 (3)	0.0002 (2)
C8	0.0162 (3)	0.0163 (3)	0.0143 (3)	−0.0005 (2)	0.0051 (2)	0.0006 (2)
C9	0.0195 (3)	0.0184 (3)	0.0152 (3)	0.0006 (2)	0.0052 (3)	0.0031 (2)
C10	0.0176 (3)	0.0193 (3)	0.0167 (3)	0.0026 (2)	0.0042 (3)	0.0029 (3)
C11	0.0154 (3)	0.0149 (3)	0.0148 (3)	−0.0003 (2)	0.0043 (2)	−0.0019 (2)
C12	0.0170 (3)	0.0169 (3)	0.0148 (3)	0.0006 (2)	0.0058 (2)	0.0016 (2)
C13	0.0158 (3)	0.0164 (3)	0.0154 (3)	0.0013 (2)	0.0049 (2)	0.0016 (2)
C14	0.0229 (3)	0.0252 (4)	0.0216 (4)	−0.0004 (3)	0.0112 (3)	0.0007 (3)
C15	0.0158 (3)	0.0350 (5)	0.0267 (4)	0.0032 (3)	0.0049 (3)	0.0048 (3)

### Geometric parameters (Å, °)

**Table:**

O1—C7	1.2346 (10)	C8—C9	1.4031 (10)
N1—C11	1.3615 (10)	C8—C13	1.4066 (11)
N1—C14	1.4494 (11)	C9—C10	1.3781 (11)
N1—C15	1.4499 (11)	C9—H9	0.9300
C1—C2	1.3924 (11)	C10—C11	1.4163 (11)
C1—C6	1.3947 (11)	C10—H10	0.9300
C1—H1	0.9300	C11—C12	1.4165 (10)
C2—C3	1.3862 (12)	C12—C13	1.3816 (11)
C2—H2	0.9300	C12—H12	0.9300
C3—C4	1.3907 (13)	C13—H13	0.9300
C3—H3	0.9300	C14—H14A	0.9600
C4—C5	1.3868 (12)	C14—H14B	0.9600
C4—H4	0.9300	C14—H14C	0.9600
C5—C6	1.3965 (11)	C15—H15A	0.9600
C5—H5	0.9300	C15—H15B	0.9600
C6—C7	1.4976 (11)	C15—H15C	0.9600
C7—C8	1.4716 (11)		
			
C11—N1—C14	121.81 (7)	C10—C9—C8	122.12 (7)
C11—N1—C15	120.55 (7)	C10—C9—H9	118.9
C14—N1—C15	116.94 (7)	C8—C9—H9	118.9
C2—C1—C6	120.15 (8)	C9—C10—C11	120.77 (7)
C2—C1—H1	119.9	C9—C10—H10	119.6
C6—C1—H1	119.9	C11—C10—H10	119.6
C3—C2—C1	120.02 (8)	N1—C11—C10	120.86 (7)
C3—C2—H2	120.0	N1—C11—C12	121.89 (7)
C1—C2—H2	120.0	C10—C11—C12	117.25 (7)
C2—C3—C4	120.36 (8)	C13—C12—C11	121.14 (7)
C2—C3—H3	119.8	C13—C12—H12	119.4
C4—C3—H3	119.8	C11—C12—H12	119.4
C5—C4—C3	119.52 (8)	C12—C13—C8	121.49 (7)
C5—C4—H4	120.2	C12—C13—H13	119.3
C3—C4—H4	120.2	C8—C13—H13	119.3
C4—C5—C6	120.76 (8)	N1—C14—H14A	109.5
C4—C5—H5	119.6	N1—C14—H14B	109.5
C6—C5—H5	119.6	H14A—C14—H14B	109.5
C1—C6—C5	119.17 (7)	N1—C14—H14C	109.5
C1—C6—C7	123.28 (7)	H14A—C14—H14C	109.5
C5—C6—C7	117.47 (7)	H14B—C14—H14C	109.5
O1—C7—C8	120.51 (7)	N1—C15—H15A	109.5
O1—C7—C6	118.26 (7)	N1—C15—H15B	109.5
C8—C7—C6	121.18 (7)	H15A—C15—H15B	109.5
C9—C8—C13	117.23 (7)	N1—C15—H15C	109.5
C9—C8—C7	117.90 (7)	H15A—C15—H15C	109.5
C13—C8—C7	124.76 (7)	H15B—C15—H15C	109.5
			
C6—C1—C2—C3	−0.63 (13)	C6—C7—C8—C13	−12.54 (12)
C1—C2—C3—C4	1.18 (13)	C13—C8—C9—C10	−0.33 (12)
C2—C3—C4—C5	−0.29 (14)	C7—C8—C9—C10	175.94 (7)
C3—C4—C5—C6	−1.15 (13)	C8—C9—C10—C11	0.20 (12)
C2—C1—C6—C5	−0.79 (12)	C14—N1—C11—C10	177.99 (7)
C2—C1—C6—C7	−177.50 (7)	C15—N1—C11—C10	7.82 (12)
C4—C5—C6—C1	1.69 (12)	C14—N1—C11—C12	−1.90 (12)
C4—C5—C6—C7	178.59 (8)	C15—N1—C11—C12	−172.07 (8)
C1—C6—C7—O1	141.72 (9)	C9—C10—C11—N1	−179.62 (7)
C5—C6—C7—O1	−35.04 (11)	C9—C10—C11—C12	0.27 (11)
C1—C6—C7—C8	−40.56 (11)	N1—C11—C12—C13	179.28 (7)
C5—C6—C7—C8	142.68 (8)	C10—C11—C12—C13	−0.61 (11)
O1—C7—C8—C9	−10.84 (12)	C11—C12—C13—C8	0.49 (12)
C6—C7—C8—C9	171.49 (7)	C9—C8—C13—C12	−0.02 (11)
O1—C7—C8—C13	165.13 (8)	C7—C8—C13—C12	−176.00 (7)

### Hydrogen-bond geometry (Å, °)

**Table:**

*D*—H···*A*	*D*—H	H···*A*	*D*···*A*	*D*—H···*A*
C4—H4···O1^i^	0.93	2.46	3.3730 (12)	168
C10—H10···Cg2^ii^	0.93	2.98	3.6452 (9)	130

Symmetry codes: (i) −*x*+1, *y*−1/2, −*z*+1/2; (ii) −*x*, *y*+1/2, −*z*+1/2.
